# Association Between Albumin‐Corrected Anion Gap and Mortality in ICU Patients With Acute Heart Failure: A MIMIC‐IV Cohort Study

**DOI:** 10.1155/cdr/9362170

**Published:** 2026-04-20

**Authors:** Huijuan Pu, Guoping Zhao, Yumin Wang, Ni An, Yan Liu, Chenyang Li, Xiuli Zhang, Jie Liu, Wanling Wu, Hong Zhu, Lei Li, Defeng Pan

**Affiliations:** ^1^ Department of General Practice, The Affiliated Hospital of Xuzhou Medical University, Xuzhou, Jiangsu, China, xzmc.edu.cn; ^2^ Department of Cardiology, The Affiliated Hospital of Xuzhou Medical University, Xuzhou, Jiangsu, China, xzmc.edu.cn; ^3^ Department of Geriatric Medicine, The Affiliated Hospital of Xuzhou Medical University, Xuzhou, Jiangsu, China, xzmc.edu.cn

**Keywords:** acute heart failure, albumin-corrected anion gap, all-cause mortality, intensive care unit

## Abstract

**Background:**

The albumin‐corrected anion gap (ACAG) has been associated with adverse outcomes in critically ill patients. However, its prognostic value in patients with acute heart failure (AHF) remains unclear. This study is aimed at investigating the association between ACAG levels and all‐cause mortality in AHF patients.

**Methods:**

This retrospective study included AHF patients admitted to the intensive care unit (ICU) for the first time, utilizing data from the Medical Information Mart for Intensive Care IV (MIMIC‐IV) database. Patients were stratified into tertiles (T1–T3) based on ACAG levels. The primary endpoints were 30‐ and 365‐day all‐cause mortality, whereas the secondary endpoints included 90‐ and 180‐day all‐cause mortality. Kaplan–Meier (K‐M) survival analyses, Cox proportional hazards models, and restricted cubic spline (RCS) analyses were employed to assess the association between ACAG and all‐cause mortality.

**Results:**

A total of 2754 patients were included, with a mean age of 74 years, and 56.5% of the participants were male. K‐M curves demonstrated that elevated ACAG levels correlated with increased mortality at 30, 90, 180, and 365 days (all log‐rank *p* < 0.001). Cox regression analysis indicated that T3 was associated with a higher risk of mortality compared with T1. RCS analysis revealed a nonlinear relationship between ACAG and all‐cause mortality. Above certain thresholds, each 1‐unit increase in ACAG was linked to a 9% increase in the risk of 30‐day mortality (hazard ratio [HR] 1.09, 95% confidence interval [CI] 1.07–1.11) and an 8% increase in the risk of 365‐day mortality (HR 1.08, 95% CI 1.06–1.09).

**Conclusion:**

ACAG levels were associated with all‐cause mortality in patients with AHF. Thus, ACAG may serve as a valuable prognostic marker for this patient population.

## 1. Introduction

Acute heart failure (AHF) is characterized by the rapid onset or exacerbation of symptoms and/or signs of heart failure, often necessitating urgent evaluation and treatment [1]. A subset of patients experiences hemodynamic instability and end‐organ dysfunction, which requires admission to the intensive care unit (ICU). The proportion of AHF patients admitted to the ICU ranges from 5% to 45.4%, as reported by various registries, and is associated with a high risk of death [2]. Despite ongoing advancements in guideline‐directed medical therapy, AHF continues to be a leading cause of unplanned hospitalizations among individuals aged 65 years and older [1, 3].

Given the elevated mortality and rehospitalization rates linked to AHF, the establishment of simple and reliable prognostic indicators is of substantial clinical importance. Consequently, this study is aimed at investigating a clinically relevant biomarker that can offer a scientific foundation for prognostic assessment and risk stratification in patients with AHF.

Acid–base imbalance is a pivotal factor in cardiovascular dysfunction and disease progression [4]. In patients with HF, metabolic acidosis often arises from tissue hypoxia, impaired perfusion, renal dysfunction, and neurohormonal changes, and it has been linked to adverse clinical outcomes [5]. The serum anion gap (AG) is commonly utilized in the evaluation of metabolic acidosis and has been shown to correlate with poor prognosis across various acute and chronic conditions [6–9]. However, AG is significantly affected by serum albumin levels, and hypoalbuminemia is prevalent among critically ill patients, which may result in an underestimation of the severity of metabolic disturbances [10].

To mitigate this limitation, Figge et al. were the first to introduce the albumin‐corrected anion gap (ACAG) as a more precise measure for assessing acid–base imbalance [11, 12]. In recent years, growing evidence from large observational studies and critical care databases has demonstrated that elevated ACAG levels are independently associated with increased mortality in critically ill populations, including patients with sepsis, acute myocardial infarction, cardiogenic shock, and acute kidney injury [13–16].

However, the prognostic significance of ACAG in critically ill patients with AHF remains uncertain. Notably, AHF exhibits distinct pathophysiological characteristics compared with other critical illnesses, and findings from diverse ICU populations cannot be directly applied to this specific subgroup. To date, no study has systematically assessed the relationship between baseline ACAG levels and mortality in ICU‐admitted patients with AHF.

Therefore, this study is aimed at evaluating the potential association between baseline ACAG levels at ICU admission and all‐cause mortality in critically ill patients with AHF. The finding of this study may help to explore new methods for early identification and improvement of prognosis in critically ill patients with AHF.

## 2. Methods

### 2.1. Data Sources

This study employed a retrospective observational design, utilizing data from the publicly accessible Medical Information Marketplace in Critical Care IV (MIMIC‐IV) database. The database includes data on patients who received inpatient care at Beth Israel Deaconess Medical Center from 2008 to 2019. As the data were de‐identified, the Institutional Review Board of Beth Israel Deaconess Medical Center waived the requirement for informed consent and approved the sharing of research resources [17]. Before data extraction, the author, H.P., obtained the necessary permissions to use the MIMIC‐IV database.

### 2.2. Study Design and Populations

**Figure 1:**
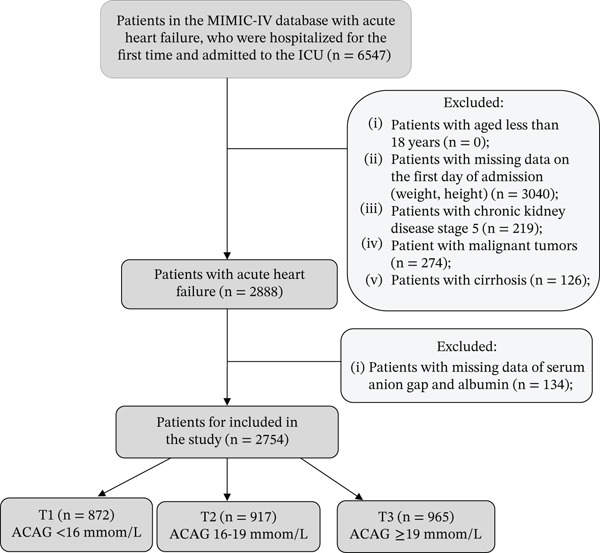
Flowchart of the selection of patients.

This study included 6547 patients who were hospitalized for the first time with AHF and admitted to the ICU. The diagnosis was based on the International Classification of Diseases, Ninth Edition (ICD‐9) and Tenth Edition (ICD‐10) codes. The ICD codes for AHF patients are provided in detail in Table S1. Patients were excluded from the study based on the following criteria: aged less than 18 years, missing data on height, weight, serum AG or albumin levels at admission, and comorbid conditions including chronic kidney disease (CKD) Stage 5, malignant tumors, or cirrhosis. After applying these exclusion criteria, 2754 patients were included in the study and divided into three groups based on tertiles of ACAG levels (Figure 1).

### 2.3. Data Extraction

In this study, extensive data for each patient at the time of admission were extracted from the database using structured query language through Navicat Premium (Version 16.1.15). The specific data extracted are shown in Table 1.

**Table 1:** Characteristics and outcomes of participants categorized by ACAG.

Variables	Total (n = 2754)	T1 (n = 872)	T2 (n = 917)	T3 (n = 965)	p
**Demographic variables**					
Age, years	74.00 (64.00, 83.00)	73.00 (62.00, 82.00)	75.00 (65.00, 84.00)	74.00 (64.00, 83.00)	0.02
Male (*n*, %)	1557 (56.54)	510 (58.49)	496 (54.09)	551 (57.10)	0.16
Race, White	1900 (68.99)	602 (69.04)	666 (72.63)	632 (65.49)	< 0.01
Weight, kg	80.00 (68.00, 96.88)	81.05 (69.00, 99.28)	82.00 (68.00, 96.20)	78.10 (67.40, 95.50)	< 0.01
BMI, kg/m^2^	28.31 (24.56, 33.12)	28.27 (24.71, 34.02)	28.73 (24.80, 33.16)	27.95 (24.20, 32.44)	< 0.01
**Past history, n (%)**					
Hypertension	810 (29.41)	317 (36.35)	277 (30.21)	216 (22.38)	< 0.01
Diabetes	1072 (38.93)	305 (34.98)	350 (38.17)	417 (43.21)	< 0.01
CHD	687 (24.95)	219 (25.11)	231 (25.19)	237 (24.56)	0.94
CKD	860 (31.23)	267 (30.62)	299 (32.61)	294 (30.47)	0.54
MI	883 (32.06)	298 (34.17)	284 (30.97)	301 (31.19)	0.27
COPD	564 (20.48)	194 (22.25)	187 (20.39)	183 (18.96)	0.22
AF	1449 (52.61)	444 (50.92)	485 (52.89)	520 (53.89)	0.44
Cardiogenic shock	532 (19.32)	103 (11.81)	162 (17.67)	267 (27.67)	< 0.01
**Medication, n (%)**					
ACEI	461 (16.74)	150 (17.20)	158 (17.23)	153 (15.85)	0.66
ARB	340 (12.35)	128 (14.68)	123 (13.41)	89 (9.22)	< 0.01
ARNI	27 (0.98)	8 (0.92)	10 (1.09)	9 (0.93)	0.92
Aspirin	2157 (78.32)	688 (78.90)	738 (80.48)	731 (75.75)	0.04
Clopidogrel	678 (24.62)	209 (23.97)	218 (23.77)	251 (26.01)	0.46
Statin	1940 (70.44)	617 (70.76)	670 (73.06)	653 (67.67)	0.04
Digoxin	383 (13.91)	121 (13.88)	121 (13.20)	141 (14.61)	0.67
Furosemid	2610 (94.77)	833 (95.53)	872 (95.09)	905 (93.78)	0.21
Spironolactone	298 (10.82)	93 (10.67)	114 (12.43)	91 (9.43)	0.11
**Laboratory data**					
RBC, K/*μ*L	3.82 (3.31, 4.30)	3.86 (3.36, 4.37)	3.82 (3.31, 4.29)	3.77 (3.28, 4.26)	0.09
WBC, K/*μ*L	10.20 (7.50, 14.00)	8.80 (6.70, 12.50)	9.80 (7.50, 13.00)	11.70 (8.80, 16.40)	< 0.01
Hemoglobin, g/dL	11.30 (9.70, 12.70)	11.40 (9.90, 12.90)	11.30 (9.80, 12.70)	11.20 (9.50, 12.60)	0.04
Platelets, K/*μ*L	207.00 (160.00, 267.00)	201.00 (160.00, 256.00)	207.00 (160.00, 270.00)	213.00 (158.00, 281.00)	0.06
RDW, %	15.90 (14.60, 17.70)	15.55 (14.40, 17.20)	15.80 (14.60, 17.30)	16.30 (14.80, 18.40)	< 0.01
Creatinine, mg/dL	1.20 (0.90, 1.70)	1.00 (0.80, 1.30)	1.10 (0.90, 1.60)	1.60 (1.10, 2.40)	< 0.01
Albumin, g/dL	3.40 (3.00, 3.80)	3.70 (3.30, 4.00)	3.40 (3.10, 3.80)	3.20 (2.80, 3.60)	< 0.01
ACAG, mmol/L	17.50 (15.25, 20.00)	14.25 (13.00, 15.00)	17.25 (16.50, 18.00)	21.25 (20.00, 23.50)	< 0.01
Glucose, mg/dL	131.00 (106.00, 174.00)	121.00 (103.00, 152.25)	128.00 (106.00, 167.00)	148.00 (112.00, 201.00)	< 0.01
Anion gap, mmol/L	15.00 (13.00, 18.00)	12.00 (11.00, 13.00)	15.00 (14.00, 16.00)	19.00 (17.00, 21.00)	< 0.01
Bicarbonate, mmol/L	24.00 (21.00, 27.00)	27.00 (24.00, 30.00)	24.00 (22.00, 27.00)	21.00 (19.00, 24.00)	< 0.01
Calcium, mg/dL	8.60 (8.20, 9.07)	8.70 (8.30, 9.10)	8.60 (8.10, 9.10)	8.50 (8.10 ,9.00)	< 0.01
Chloride, mEq/L	102.00 (98.00, 105.00)	103.00 (99.00, 106.00)	102.00 (99.00, 106.00)	101.00 (97.00, 105.00)	< 0.01
Sodium, mEq/L	139.00 (136.00, 141.00)	139.00 (137.00, 141.00)	139.00 (136.00, 141.00)	138.00 (135.00, 141.00)	< 0.01
Potassium, mEq/L	4.20 (3.80, 4.60)	4.20 (3.80, 4.50)	4.10 (3.80, 4.50)	4.30 (3.90, 4.80)	< 0.01
AST, IU/L	47.00 (27.00, 128.75)	38.00 (25.00, 75.00)	45.00 (25.00, 110.00)	66.00 (33.00, 242.00)	< 0.01
ALT, IU/L	36.00 (20.00, 97.00)	30.00 (19.00, 65.25)	33.00 (19.00, 78.00)	49.00 (24.00, 164.00)	< 0.01
APSIII	53.00 (39.00, 72.00)	52.00 (40.00, 72.00)	51.00 (38.00, 69.00)	55.00 (40.00, 74.00)	0.04
SAPSII	41.00 (33.00, 50.00)	41.00 (33.00, 50.00)	40.00 (33.00, 50.00)	41.00 (33.00, 51.00)	0.60
SOFA	6.00 (4.00, 10.00)	7.00 (4.00, 10.00)	6.00 (4.00, 9.00)	6.00 (4.00, 10.00)	0.19
OASIS	35.00 (29.00, 42.00)	35.00 (28.00, 42.00)	34.00 (28.00, 41.00)	35.00 (29.00, 42.00)	0.10
**Length of stay (LOS), day**					
LOS hospital	10.88 (6.88, 16.49)	10.37 (6.69, 15.66)	10.98 (7.03, 16.03)	11.17 (6.97, 17.95)	0.06
LOS ICU	3.46 (1.96, 6.40)	3.16 (1.77, 5.74)	3.40 (1.98, 6.15)	3.91 (2.08, 7.23)	< 0.01
**Outcomes, n (%)**					
In‐hospital mortality	332 (12.06)	58 (6.65)	96 (10.47)	178 (18.45)	< 0.01
30‐day mortality	506 (18.37)	105 (12.04)	139 (15.16)	262 (27.15)	< 0.01
90‐day mortality	654 (23.75)	151 (17.32)	180 (19.63)	323 (33.47)	< 0.01
180‐day mortality	764 (27.74)	181 (20.76)	213 (23.23)	370 (38.34)	< 0.01
365‐day mortality	891 (32.35)	223 (25.57)	253 (27.59)	415 (43.01)	< 0.01

Abbreviations: ACAG, albumin‐corrected anion gap; ACEI, angiotensin‐converting enzyme inhibitor; AF, atrial fibrillation; ALT, alanine aminotransferase; APSIII, acute physiology score III; ARB, angiotensin receptor blockers; ARNI, angiotensin receptor‐neprilysin inhibitor; AST, aspartate aminotransferase; BMI, body mass index; CHD, coronary heart disease; CKD, chronic kidney disease; COPD, chronic obstructive pulmonary disease; MI, myocardial infarction; OASIS, Oxford Acute Severity Of Illness Score; RBC, red blood cell; RDW, red blood cell distribution width; SAPS II, Simplified Acute Physiology Score; SOFA, sequential organ failure assessment; WBC, white blood cell.

Clinical data were extracted from the first measurement within 24 h after admission to the ICU, and variables with more than 20% missing values were excluded, whereas those with less than 20% missing values were imputed using multiple imputation by chained equations to ensure data completeness and the accuracy of the analysis.

The primary outcome of this study was 30‐ and 365‐day all‐cause mortality. The secondary outcome was 90‐ and 180‐day all‐cause mortality.

### 2.4. Calculation of ACAG

*AG (mmol/l)* = *(sodium [mmol/L]* + *potassium [mmol/L])* ‐ *(chloride [mmol/L]* + *bicarbonate [mmol/L])*, and *A* *C* *A* *G* = *A* *G* + (4.4–*a* *l* *b* *u* *m* *i* *n* [*g*/*d* *L*])∗ 2.5 [14].

### 2.5. Statistical Analysis

Continuous variables were expressed as means ± standard deviation or as medians with interquartile ranges (IQR). Categorical variables were analyzed using the chi‐square test or Fisher′s exact test and are presented as absolute values and percentages. The incidence of primary and secondary outcomes was determined based on stratification by ACAG tertiles, using Kaplan–Meier (K‐M) curves. Univariate Cox regression analysis was employed to assess the association between ACAG and 30‐, 90‐, 180‐, and 365‐day mortality. Multivariate Cox proportional hazards regression models incorporated variables that were clinically relevant or had a univariate association with the outcome.

Three models were constructed for analysis: Crude model included only ACAG. Model 1 was adjusted for age, gender, race, and body mass index (BMI). Model 2 was further adjusted for hypertension, diabetes, use of angiotensin receptor blockers (ARB), aspirin, and statins, as well as laboratory parameters including white blood cell count (WBC), hemoglobin (Hb), red blood cell distribution width (RDW), creatinine, alanine aminotransferase (ALT), aspartate aminotransferase (AST), and Acute Physiology Score III (APSIII). The lowest tertile of ACAG was used as the reference category in all three models.

ACAG was further analyzed as a continuous variable using restricted cubic splines (RCS) to explore dose–response relationships with the risk of primary and secondary outcomes. If the correlation was nonlinear, inflection points between the ACAG and 30‐, 90‐, 180‐, and 365‐day mortality were identified using a recursive algorithm. To further examine the relationship between ACAG and 30‐, 90‐, 180‐, and 365‐day mortality, a two‐segment Cox proportional hazards model was applied on either side of the inflection point. Additionally, stratified analyses were performed based on gender, age (< 60 or ≥ 60 years), race, hypertension, diabetes, chronic obstructive pulmonary disease (COPD), and CKD. All statistical analyses were conducted using SPSS software (Version 27.0) and R Studio (Version 4.3.1), with *p* values of < 0.05 considered statistically significant.

## 3. Results

### 3.1. Baseline Characteristics

A total of 2754 patients with AHF were included and stratified into ACAG tertiles (T1: < 16; T2: 16–19; and T3: ≥ 19 mmol/L).

Patients in the high‐ACAG tertile group had a lower prevalence of hypertension but a higher prevalence of diabetes and cardiogenic shock. The use of ARB was less frequent in this group. Higher ACAG levels were associated with laboratory features indicative of systemic inflammation, metabolic disturbance, and organ dysfunction, including elevated WBC, RDW, creatinine, glucose, AST, ALT, and APSIII scores, along with lower Hb, albumin, electrolytes, and bicarbonate levels. Moreover, as the ACAG level increases, patients spend a longer time in the ICU, and their mortality rates during hospitalization and during follow‐up periods (30‐, 90‐, 180‐, and 365‐day) are also higher (Table 1).

### 3.2. Study Outcome

K‐M survival curve analysis revealed significant differences in mortality rates at 30‐ and 365‐day among patients stratified by ACAG tertiles (Figure 2), as well as at 90‐ and 180‐day (Figure S1). Specifically, compared with patients with lower ACAG levels, those with the highest ACAG levels had significantly lower survival rates at 30‐, 90‐, 180‐, and 365‐day (Log‐rank *p* < 0.001).

Figure 2Kaplan–Meier survival curves for 30‐ (a) and 365‐day (b) all‐cause mortality according to tertiles of ACAG in ICU patients with AHF.

**Figure:**
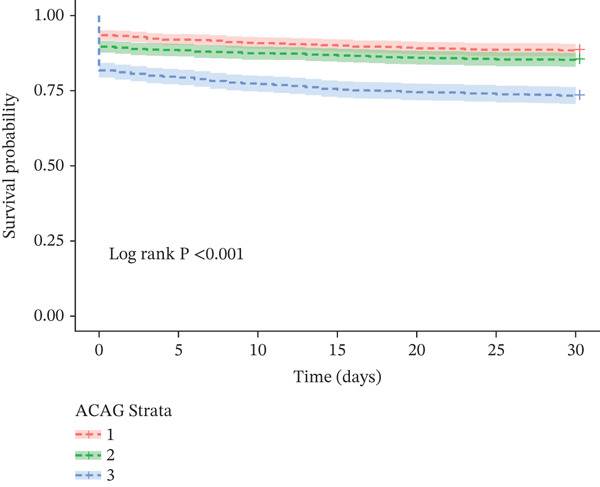
(a)

**Figure:**
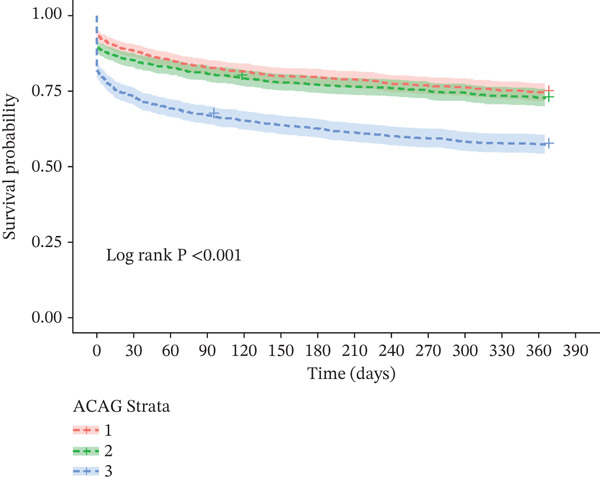
(b)

### 3.3. Relationship Between ACAG and Clinical Outcomes in AHF Patients

Cox proportional hazards regression was performed to evaluate the association between ACAG levels and all‐cause mortality, using the lowest tertile as the reference. In the crude model, patients in the highest ACAG tertile had significantly increased risks of 30‐ (HR 2.49, 95% CI 1.98–3.12) and 365‐day mortality (HR 1.96, 95% CI 1.67–2.31). The associations remained significant after adjustment for demographic factors (Model 1) and further clinical and laboratory variables (Model 2). In the fully adjusted model, the highest tertile was independently associated with increased 30‐ (HR 1.87, 95% CI 1.46–2.38) and 365‐day mortality (HR 1.50, 95% CI 1.26–1.80). Similar findings were observed for 90‐ and 180‐day mortality (Table 2).

**Table 2:** Cox proportional hazard models for albumin‐corrected anion gap and all‐cause mortality.

Serum anion gap			
	T1	T2	T3
**30-day mortality**			
Number of deaths (%)	105 (12.04)	139 (15.16)	262 (27.15)
Crude model HR (95% CI) *p* value	Ref	1.29 (1.00, 1.67) *p* = 0.05	2.49 (1.98, 3.12) < 0.01
Model 1 HR (95% CI) *p* value	Ref	1.23 (0.95, 1.59) *p* = 0.11	2.39 (1.90, 2.99) < 0.01
Model 2 HR (95% CI) *p* value	Ref	1.18 (0.91, 1.52) *p* = 0.21	1.87 (1.46, 2.38) < 0.01
**90-day mortality**			
Number of deaths (%)	151 (17.32)	180 (19.63)	323 (33.47)
Crude model HR (95% CI) *p* value	Ref	1.17 (0.94, 1.45) *p* = 0.16	2.19 (1.80, 2.65) < 0.01
Model 1 HR (95% CI) *p* value	Ref	1.11 (0.89, 1.38) *p* = 0.35	2.12 (1.75, 2.57) < 0.01
Model 2 HR (95% CI) *p* value	Ref	1.07 (0.86, 1.32) *p* = 0.57	1.70 (1.37, 2.09) < 0.01
**180-day mortality**			
Number of deaths (%)	181 (20.76)	213 (23.23)	370 (38.34)
Crude model HR (95% CI) *p* value	Ref	1.15 (0.94, 1.40) *p* = 0.16	2.12 (1.78, 2.53) < 0.01
Model 1 HR (95% CI) *p* value	Ref	1.09 (0.90, 1.33) *p* = 0.37	2.07 (1.73, 2.47) < 0.01
Model 2 HR (95% CI) *p* value	Ref	1.05 (0.86, 1.29) *p* = 0.61	1.66 (1.37, 2.02) < 0.01
**365-day mortality**			
Number of deaths (%)	223 (25.57)	253 (27.59)	415 (43.01)
Crude model HR (95% CI) *p* value	Ref	1.11 (0.93, 1.33) p = 0.26	1.96 (1.67, 2.31) < 0.01
Model 1 HR (95% CI) *p* value	Ref	1.05 (0.88, 1.26) *p* = 0.57	1.93 (1.64, 2.27) < 0.01
Model 2 HR (95% CI) *p* value	Ref	1.01 (0.84, 1.21) *p* = 0.96	1.50 (1.26, 1.80) < 0.01

*Note:* Crude Model: Univariate model. Model 1: adjusted for age, gender, and race, BMI. Model 2: adjusted for age, gender, BMI, race, hypertension, diabetes, ARB, aspirin, statin, WBC, Hb, RDW, creatinine, ALT, AST, and APSIII.

### 3.4. Detection of Nonlinear Relationships

The RCS analysis revealed a nonlinear relationship between ACAG levels and all‐cause mortality at 30‐, 90‐, 180‐, and 365‐day mortality in AHF patients (*p* for nonlinear < 0.001) (Figures 3 and S2). To further explore this nonlinear, researchers combined the Cox proportional hazards model with a two‐piecewise Cox proportional hazards model, which showed statistical significance (*p* for likelihood ratio test < 0.05). The identified inflection points were 12.25, 12.75, 12.73, and 12.26 mmol/L for 30‐, 90‐, 180‐, and 365‐day mortality, respectively. Above these thresholds, for every one unit increase in ACAG, the risk of death increased by approximately 8% within 90, 180, and 365 days, and by about 9% within 30 days (Table 3).

Figure 3Restricted cubic spline regression analysis of the association between ACAG and all‐cause mortality in ICU patients with AHF (a) 30‐ and (b) 365‐day mortality.

**Figure:**
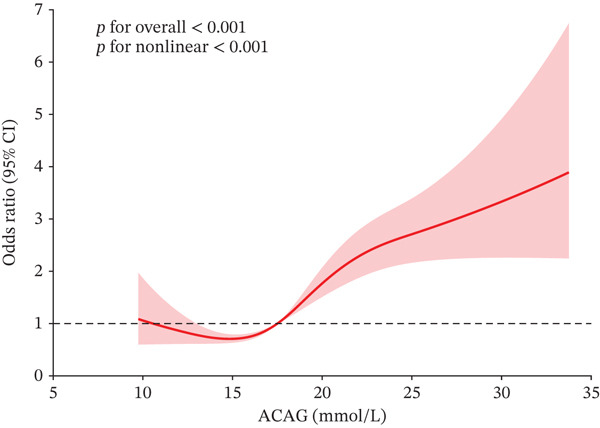
(a)

**Figure:**
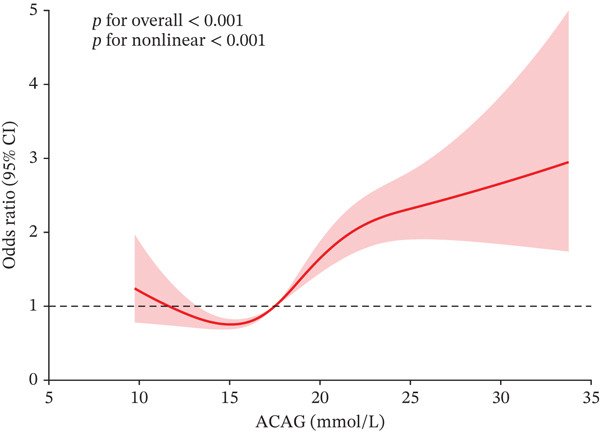
(b)

**Table 3:** Threshold effect of ACAG on all‐cause mortality in AHF patients.

	HR (95%CI)	HP‐Value
**30**‐day **mortality**		
Model 1: Fitting model by standard linear regression	1.09 (1.07–1.10)	< 0.01
Model 2: Fitting model by two‐piecewise linear regression		
Inflection point	12.25	
< 12.25	0.72 (0.53–0.98)	0.03
≥ 12.25	1.09 (1.07–1.11)	< 0.01
*p* for likelihood test		< 0.01
**90**‐**day mortality**		
Model 1: Fitting model by standard linear regression	1.08 (1.06–1.09)	< 0.01
Model 2: Fitting model by two‐piecewise linear regression		
Inflection point	12.75	
< 12.75	0.74 (0.58–0.94)	0.01
≥ 12.75	1.08 (1.07–1.10)	< 0.01
*p* for likelihood test		< 0.01
**180**‐**day mortality**		
Model 1: Fitting model by standard linear regression	1.07 (1.06–1.09)	< 0.01
Model 2: Fitting model by two‐piecewise linear regression		
Inflection point	12.73	
< 12.73	0.75 (0.59–0.95)	0.02
≥ 12.73	1.08 (1.06–1.10)	< 0.01
*p* for likelihood test		0.01
**365-day mortality**		
Model 1: Fitting model by standard linear regression	1.07 (1.05–1.08)	< 0.01
Model 2: Fitting model by two‐piecewise linear regression		
Inflection point	12.26	
< 12.26	0.71 (0.57–0.89)	< 0.01
≥ 12.26	1.08 (1.06–1.09)	< 0.01
*p* for likelihood test		< 0.01

### 3.5. Subgroup Analysis

Subgroup analyses for 30‐, 90‐, 180‐, and 365‐day all‐cause mortality demonstrated that the positive association between ACAG and mortality remained generally consistent across most predefined subgroups. Elevated ACAG was significantly associated with increased 30‐day mortality in both males and females, patients aged ≥ 60 years, White and non‐White populations, as well as in patients with or without COPD, without diabetes, and without hypertension.

**Figure 4:**
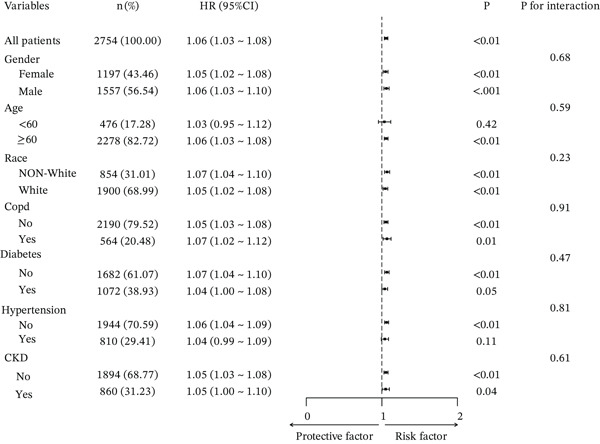
Forest plot of subgroup analyses evaluating the association between ACAG and 30‐day all‐cause mortality in ICU patients with AHF.

**Figure 5:**
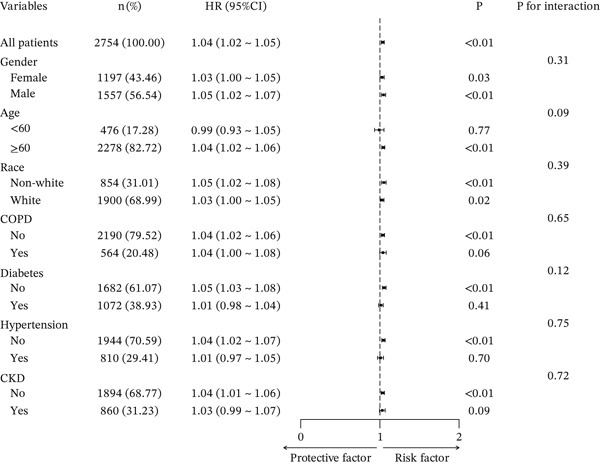
Forest plot of subgroup analyses evaluating the association between ACAG and 365‐day all‐cause mortality in ICU patients with AHF.

Although the association did not reach statistical significance in patients aged < 60 years or in those with hypertension, no significant interaction effects were observed across subgroups (all *p* for interaction > 0.05). These findings suggest that the prognostic impact of ACAG on short‐term mortality was stable and not significantly modified by demographic characteristics or major comorbidities. (Figures 4, 5, S3, and S4).

## 4. Discussion

This study represents the first investigation into the relationship between ACAG levels and short‐ and long‐term all‐cause mortality in ICU patients with AHF.

We demonstrated that elevated ACAG levels were independently associated with increased risks of mortality at 30, 90, 180, and 365 days, following comprehensive adjustments for demographic characteristics, comorbidities, laboratory parameters, and disease severity scores. Furthermore, RCS analysis revealed a nonlinear relationship between ACAG and mortality, indicating a threshold effect beyond which the risk of mortality increased progressively. These findings suggest that ACAG may serve as a significant prognostic indicator in critically ill patients with AHF.

These findings align with previous research indicating that elevated AG predicts adverse outcomes in HF and other critical conditions. However, conventional AG does not consider serum albumin, which substantially influences its value. Hypoalbuminemia is prevalent among critically ill patients and may result in the underestimation of unmeasured anions when uncorrected AG is utilized [18]. In our cohort, over half of the patients (50.18%) presented with hypoalbuminemia upon admission. By incorporating albumin correction, the ACAG may provide a more accurate representation of metabolic acidosis and the burden of unmeasured anions. Although ACAG has been associated with mortality in sepsis, acute myocardial infarction, cardiogenic shock, and acute kidney injury, evidence in AHF has been lacking [13, 19–22]. This study therefore extends existing evidence to this high‐risk population.

The nonlinear relationship observed in our analysis further refines risk stratification. To the right of the threshold, a positive correlation was observed between ACAG levels and all‐cause mortality. Conversely, to the left of the threshold, a negative correlation was observed between ACAG levels and all‐cause mortality. This may be attributed to several factors. First, lower ACAG levels may reflect better acid–base balance and electrolyte homeostasis, supporting more stable metabolic and physiological functions, which in turn could positively influence patient prognosis. Second, unaccounted confounding factors may obscure the true relationship between ACAG levels and mortality.

The association between elevated ACAG and mortality in AHF is likely multifactorial. In critically ill patients with AHF, reduced cardiac pumping function often leads to insufficient blood supply to systemic tissues and organs, resulting in anaerobic respiration and subsequent lactate accumulation [23]. However, lactate levels can be influenced by various factors including renal disease, neurohormonal and inflammation, and so on. Renal dysfunction may further impair renal acid excretion, leading to the accumulation of organic and inorganic acids [24]. In addition, neurohormonal activation and systemic inflammation exacerbate metabolic and electrolyte disturbances. Serum albumin level reflects inflammation, malnutrition, hepatic dysfunction, and overall disease severity. Thus, ACAG may be an integrative marker of AHF‐related metabolic.

In subgroup analyses, the association between ACAG and mortality remained generally consistent across most predefined subgroups. Although statistical significance was not observed in patients younger than 60 years or in those with hypertension, no significant interaction effects were detected. The lack of significance in younger patients may be attributable to their relatively smaller sample size and greater physiological reserve, which could mitigate the impact of metabolic disturbances on short‐term outcomes. For patients with hypertension, long‐term vascular adaptation and medical therapy may partially modify the relationship between metabolic abnormalities and prognosis. Nonetheless, no significant interaction effect was detected; hypertension status did not appear to significantly modify the association between ACAG and mortality. Nonetheless, these findings require further validation in independent cohorts.

From a clinical perspective, ACAG is derived from routinely available laboratory parameters and is inexpensive and easily accessible. Therefore, it may serve as a readily obtainable prognostic marker to assist in early risk assessment in critically ill patients with AHF; however, its clinical utility for guiding decision‐making requires further validation.

This study has several limitations. First, based on observational data extracted from the MIMIC‐IV database, causal relationships are difficult to establish. Second, despite adjusting for various variables and conducting subgroup analyses, we cannot completely rule out the potential impact of unmeasured confounding factors on the outcomes. Third, the current study investigated the relationship between ACAG and mortality based on a single ACAG measurement. Therefore, further research is needed to explore the dynamic changes in ACAG and their relationship with mortality in these patients.

In conclusion, elevated ACAG levels were independently and nonlinearly associated with increased short‐ and long‐term mortality in ICU patients with AHF. Therefore, ACAG levels may serve as an early indicator of adverse outcomes in AHF patients. Clinicians should be aware that elevated ACAG levels may identify patients at higher risk, who may benefit from closer monitoring and comprehensive evaluation.

## Author Contributions

H.P.: conceptualization, methodology, writing—original draft, data analysis. G.Z., Y.W., N.A., Y.L., and C.L.: data reduction and analysis, methodology. X.Z., J.L., W.W., H.Z., and L.L.: writing—review and editing, supervision. D.P. guided the analysis and made substantial improvements to the paper.

## Funding

No funding was received for this manuscript.

## Conflicts of Interest

The authors declare no conflicts of interest.

## Supporting Information

Additional supporting information can be found online in the Supporting Information section.

## Supporting information


**Supporting Information 1** Table S1: AHF ICD‐code.

**Supporting Information 2** Figure S1: Kaplan–Meier survival curves for 90‐ (a) and 180‐day (b) all‐cause mortality according to tertiles of ACAG in ICU patients with AHF.

**Supporting Information 3** Figure S2: Restricted cubic spline regression analysis of the association between ACAG and all‐cause mortality in ICU patients with AHF (a) 90‐ and (b) 180‐day mortality.

**Supporting Information 4** Figure S3: Forest plot of subgroup analyses evaluating the association between ACAG and 90‐day all‐cause mortality in ICU patients with AHF.

**Supporting Information 5** Figure S4: Forest plot of subgroup analyses evaluating the association between ACAG and 180‐day all‐cause mortality in ICU patients with AHF.

## Data Availability

The original data supporting the conclusions of this paper can be reasonably obtained from the corresponding authors.
