# Supplementary material for: Association Between Albumin‐Corrected Anion Gap and Mortality in ICU Patients With Acute Heart Failure: A MIMIC‐IV Cohort Study
Source: Cardiovasc Ther. 2026 Apr 20;2026:9362170. doi: 10.1155/cdr/9362170 (PMC13093543; doi:10.1155/cdr/9362170)
Supplement: Supplementary file 1 — Supporting Information 1 Table S1: AHF ICD‐code. [file CDR-2026-9362170-s004.docx]

| AHF ICD-Code | |
| --- | --- |
| ICD-9 | 42821, 42823, 42831, 42833, 42841, 42843 |
| ICD-10 | I5021, I5023, I5031, I5033, I5043 |

Supplementary Table 1
