# Supplementary figures and images for: Association Between Albumin‐Corrected Anion Gap and Mortality in ICU Patients With Acute Heart Failure: A MIMIC‐IV Cohort Study
Source: Cardiovasc Ther. 2026 Apr 20;2026:9362170. doi: 10.1155/cdr/9362170 (PMC13093543; doi:10.1155/cdr/9362170)

ACAG Strata 1 2 3

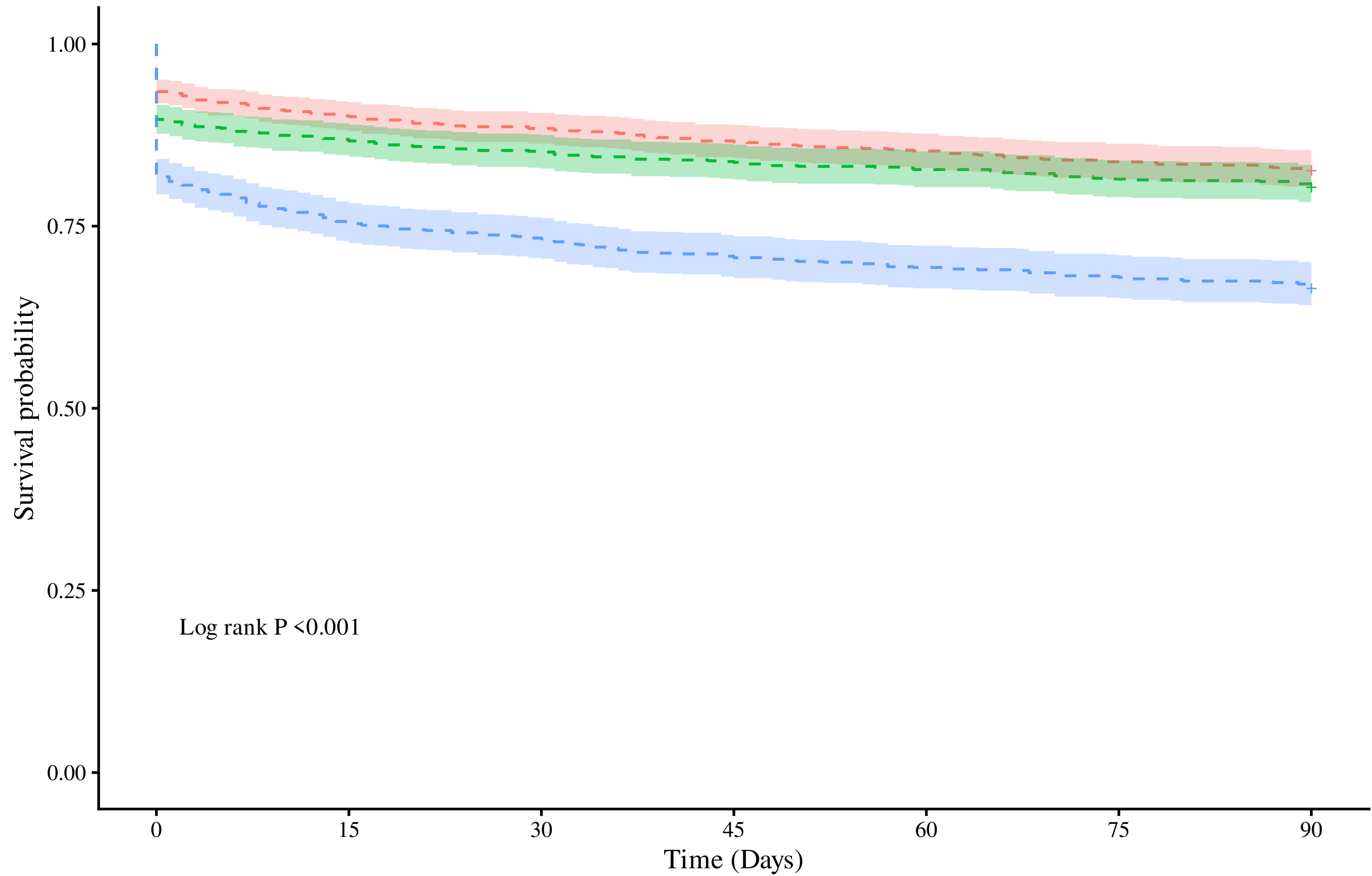

Supplement: Supplementary file 2 — Supporting Information 2 Figure S1: Kaplan–Meier survival curves for 90‐ (a) and 180‐day (b) all‐cause mortality according to tertiles of ACAG in ICU patients with AHF. [file CDR-2026-9362170-s003.zip › 9362170.f2/Supplementary Figure 1 KM-curve (A).pdf]

ACAG Strata 1 2 3

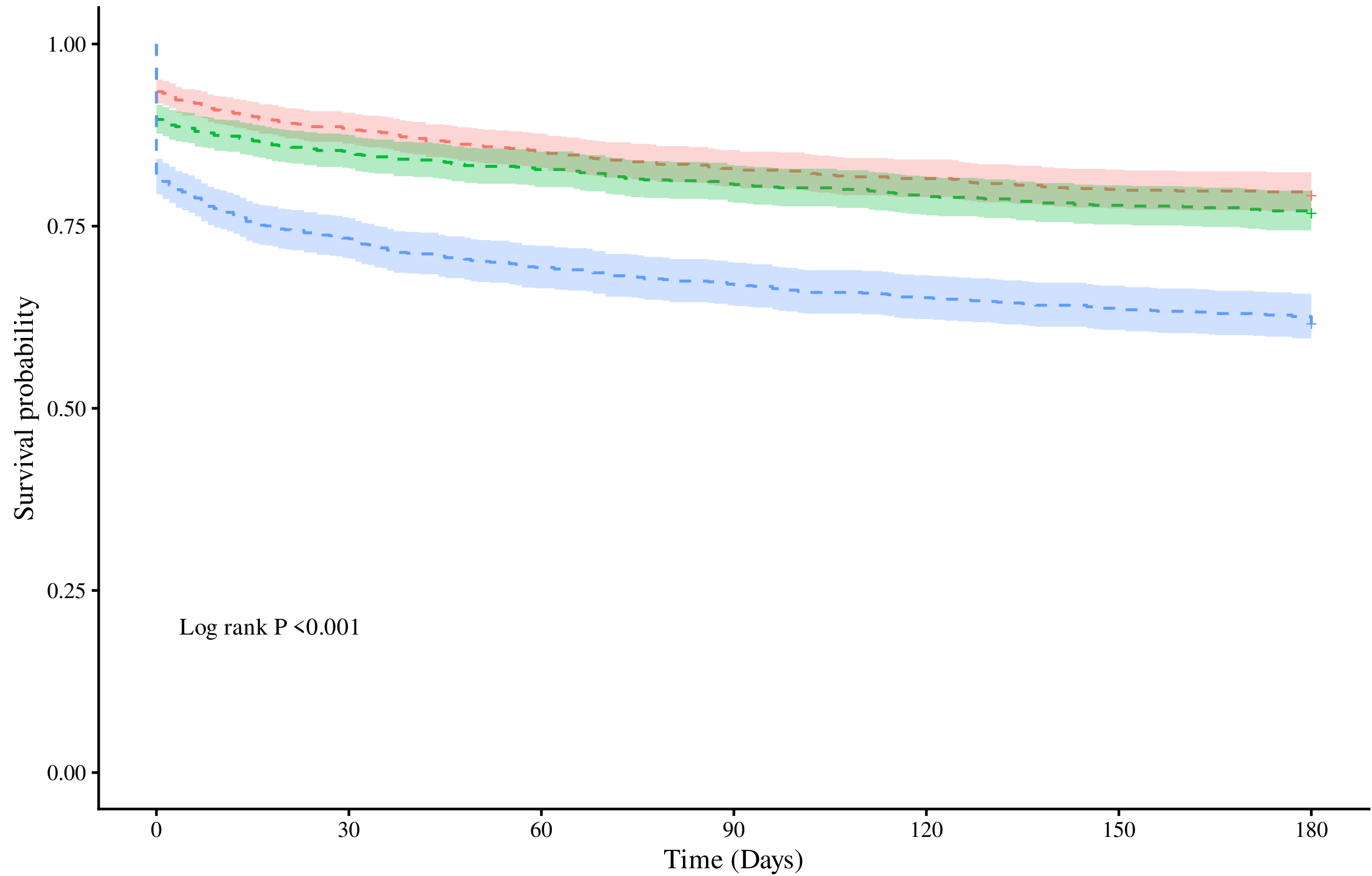

Supplement: Supplementary file 2 — Supporting Information 2 Figure S1: Kaplan–Meier survival curves for 90‐ (a) and 180‐day (b) all‐cause mortality according to tertiles of ACAG in ICU patients with AHF. [file CDR-2026-9362170-s003.zip › 9362170.f2/Supplementary Figure 1 KM-curve (B).pdf]

P for overall < 0.001  
P for nonlinear < 0.001

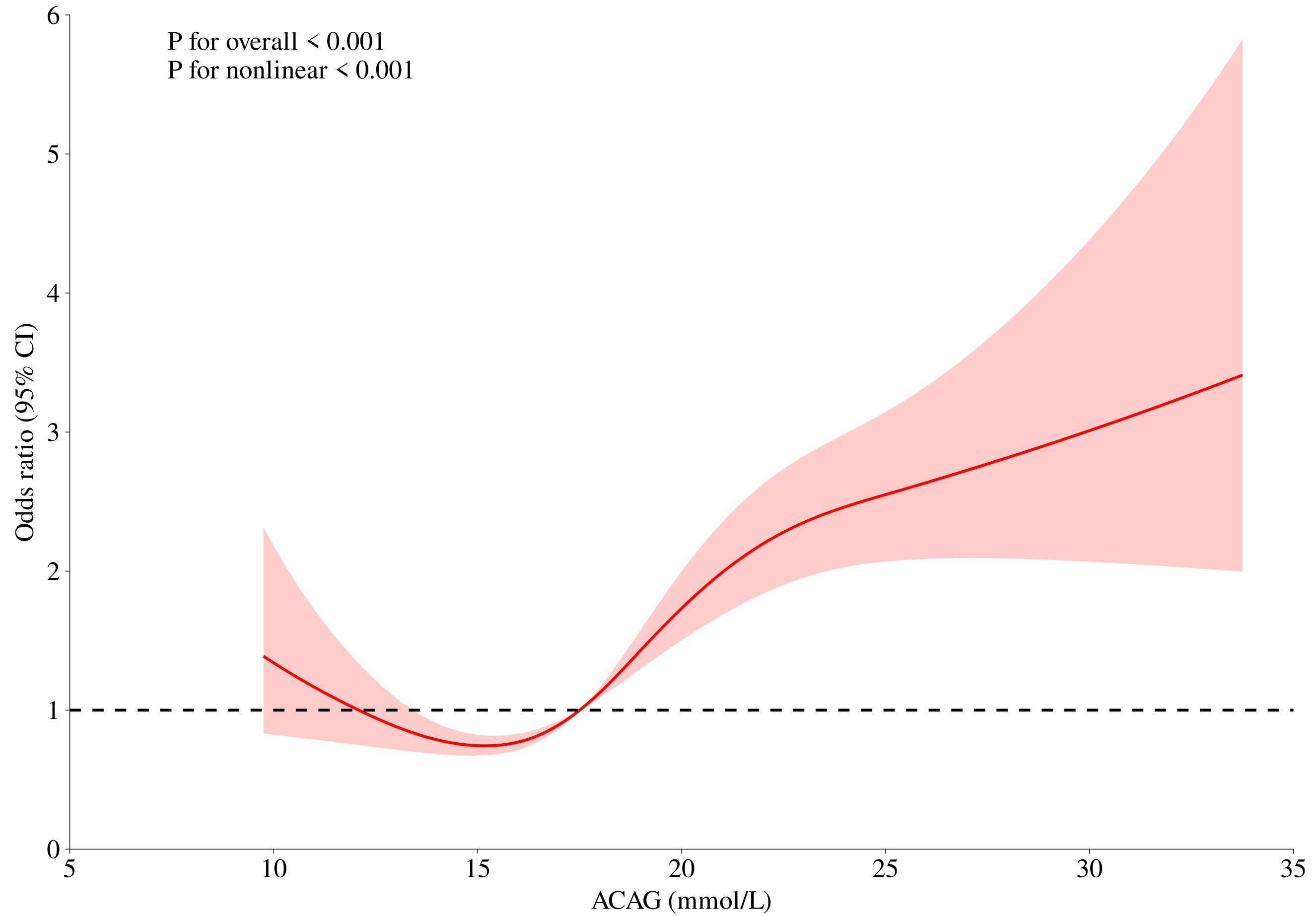

Supplement: Supplementary file 3 — Supporting Information 3 Figure S2: Restricted cubic spline regression analysis of the association between ACAG and all‐cause mortality in ICU patients with AHF (a) 90‐ and (b) 180‐day mortality. [file CDR-2026-9362170-s002.zip › 9362170.f3/Supplementary Figure 2 RCS (A).pdf]

P for overall < 0.001  
P for nonlinear < 0.001

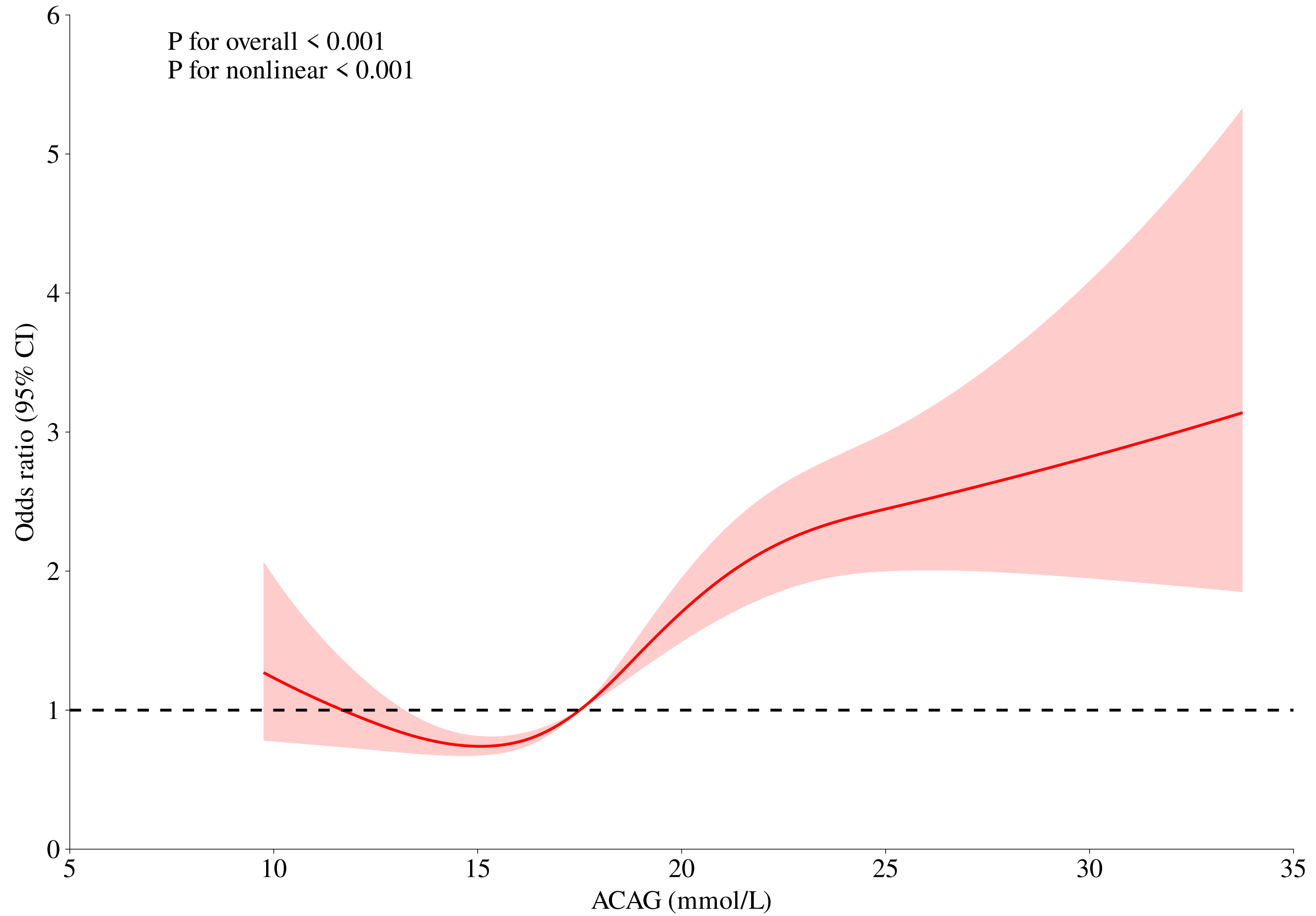

Supplement: Supplementary file 3 — Supporting Information 3 Figure S2: Restricted cubic spline regression analysis of the association between ACAG and all‐cause mortality in ICU patients with AHF (a) 90‐ and (b) 180‐day mortality. [file CDR-2026-9362170-s002.zip › 9362170.f3/Supplementary Figure 2 RCS (B).pdf]

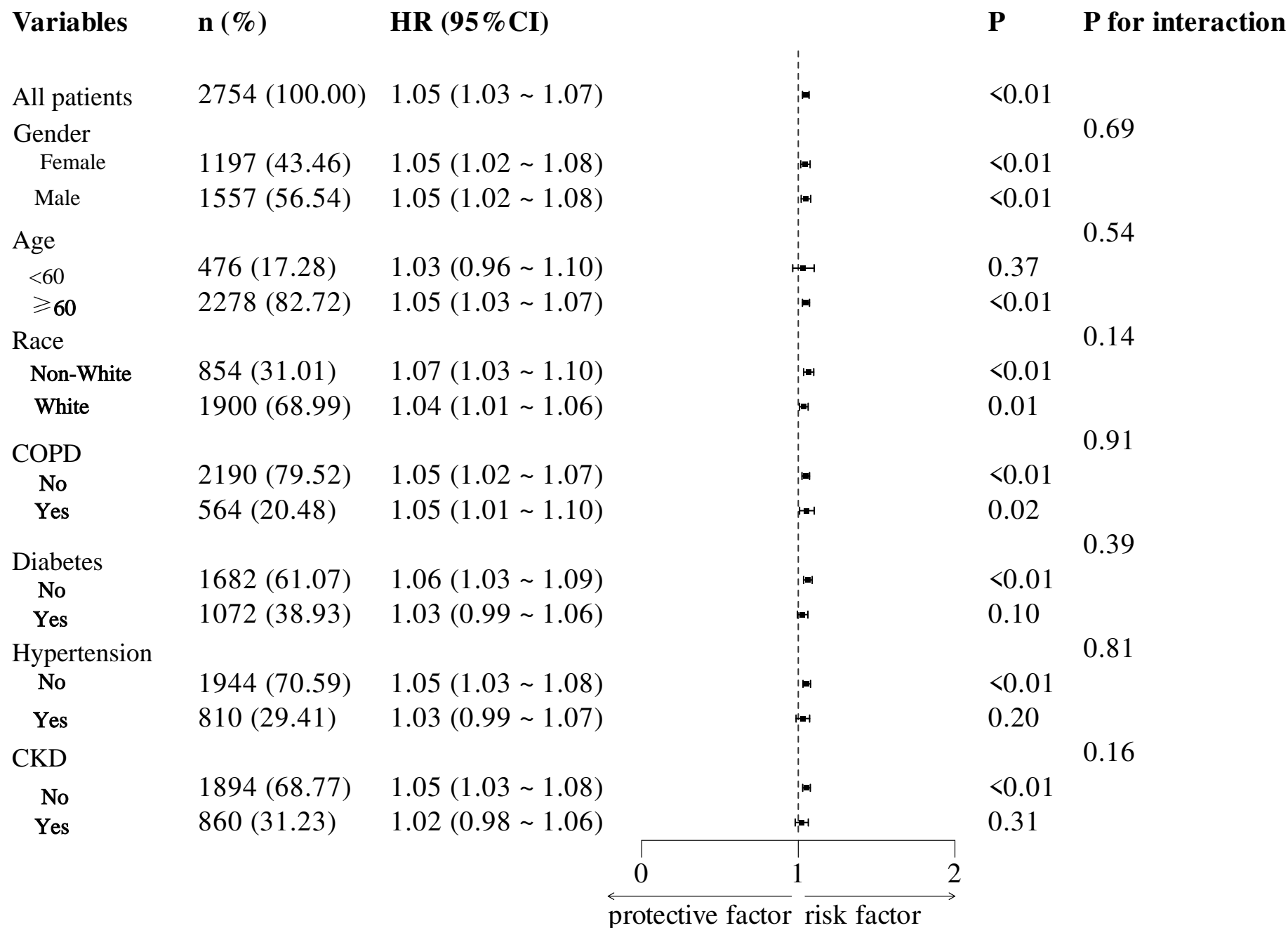

**Supplementary figure 3** Forest plots of stratified analyses of ACAG and 90-day all-cause mortality

Supplement: Supplementary file 4 — Supporting Information 4 Figure S3: Forest plot of subgroup analyses evaluating the association between ACAG and 90‐day all‐cause mortality in ICU patients with AHF. [file CDR-2026-9362170-s001.pdf]

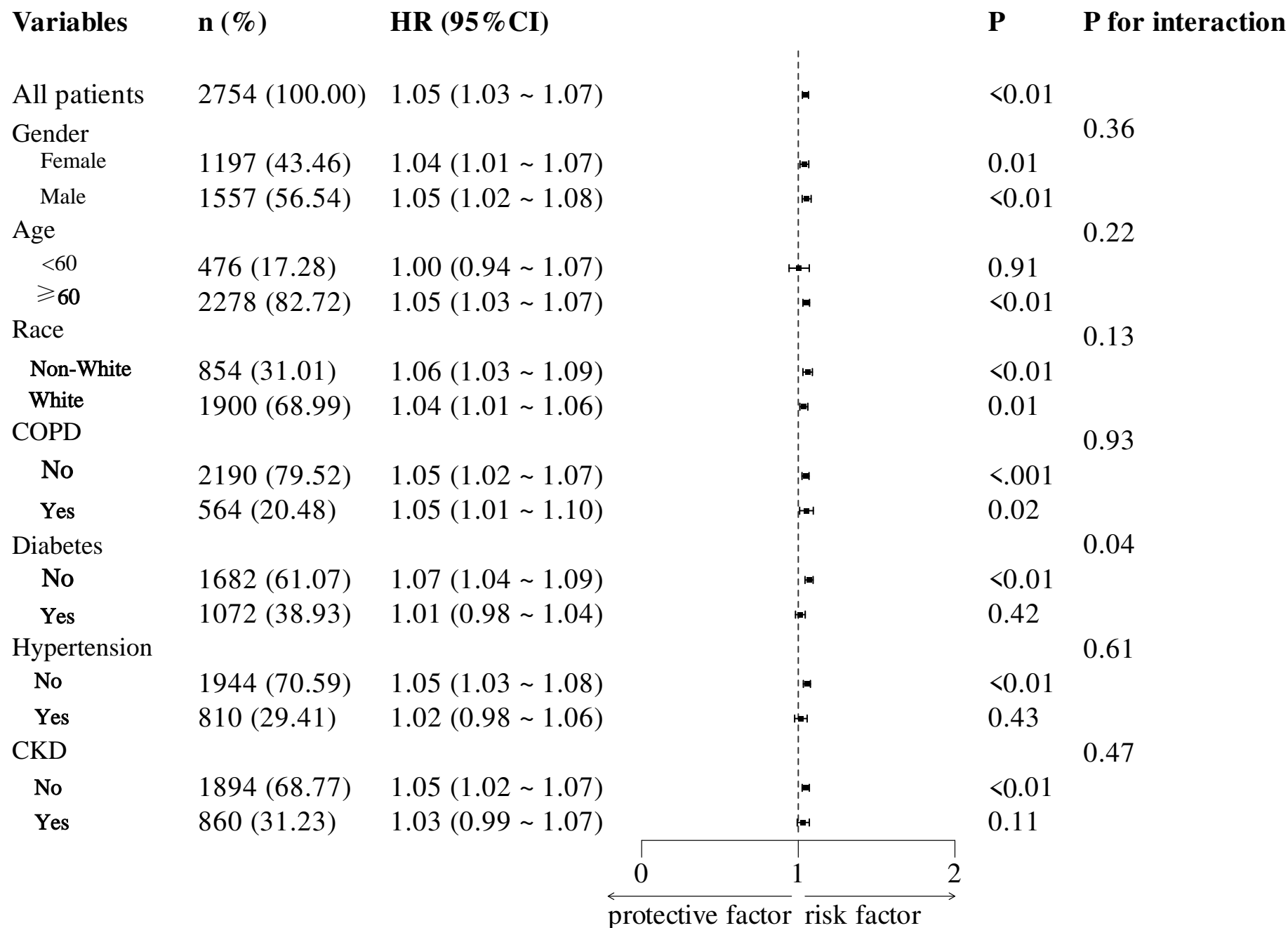

**Supplementary figure 4** Forest plots of stratified analyses of ACAG and 180-day all-cause mortality

Supplement: Supplementary file 5 — Supporting Information 5 Figure S4: Forest plot of subgroup analyses evaluating the association between ACAG and 180‐day all‐cause mortality in ICU patients with AHF. [file CDR-2026-9362170-s005.pdf]
